# Modelling Gender Differences in the Economic and Social Influences of Obesity in Australian Young People

**DOI:** 10.3390/ijerph14030257

**Published:** 2017-03-03

**Authors:** Gulay Avsar, Roger Ham, W. Kathy Tannous

**Affiliations:** School of Business, Western Sydney University, Sydney 2751, Australia; g.avsar@westernsydney.edu.au (G.A.); rogham@gmail.com (R.H.)

**Keywords:** obesity, youth, gender, panel model, policy

## Abstract

In Australia, as in many other developed economies, the prevalence of obesity has risen significantly in all age groups and especially in young males and females over the past decade. Using data from the Household, Income and Labour Dynamics in Australia (HILDA) Survey, this paper investigates the influence of economic, personality and social factor demographics on the incidence of obesity in Australian youths. The study uses two random parameters logit models, including one that allows for gender-specific differences in the conditioning variables. The models reveal notable differences between the most important variables affecting the incidence of obesity amongst females compared to males. These differences are notable to consider for policy and intervention programs aimed at reducing the problem of obesity.

## 1. Introduction

Obesity has been said to constitute a global ”crisis” [1,2,3,4,5]—this is increasingly the case among children and young people [6,7,8,9,10]. In the U.S. for instance, 32.6% of boys aged 12 to 19 years, and 29.9% of girls within this age cohort have a high body mass index (BMI), at or above the 85th percentile [11]. Using measured height and weight data during a five-year transitional period, another longitudinal study in the U.S. found that a high proportion of adolescents became and remained obese into adulthood [12]. Similarly in England, 21% of boys aged 11 to 15 years, and 18% of girls of this age are considered obese [13]. In Canada, self-reported rates of obesity are considerably less, with 6.8% of boys aged 12 to 17 years, and 2.9% of girls of this age cohort deemed to be obese; however, the bona fide rates are estimated to be higher [14].

In Australia, the prevalence of obesity has risen significantly in all age groups and especially young males and females over the past several decades [15]. According to the Australian Institute of Health and Welfare (AIHW) 2011 report, 35% of young people aged 12 to 24 years are overweight or obese. Excessive body weight among children and young people has considerable implications at individual, social and economic levels [16]. At an individual level, young people who are overweight are at increased risk of co-morbidities including type 2 diabetes [17], hypertension, respiratory problems, including asthma and obstructive sleep apnea [18], as well as affective disorders, including depression and anxiety [19]. Even before these clinical indicators manifest, overweight young people are likely to experience poor self-esteem and engage in at-risk behaviours including tobacco and alcohol use [20]. Beyond their youth, children and young people who are overweight or obese are likely to experience ill effects as they enter adulthood [21,22], including chronic health issues [23].

Weight and weight gain in children, young people and older adults have been linked to personality traits as defined by the five-factor model (FFM) [24,25]. Likewise, weight has been associated with personality trait development in adulthood and children [25,26]. In the development of intervention and prevention plans to address obesity in young people, greater evidence on relationship of personality traits by gender differences is needed [27].

Excessive body weight among children and young people also represents a considerable drain on the public purse. Poor wellbeing is likely to warrant more services, more hospitalisation, more treatment, more medication, and continued access to clinicians and practitioners [28]. Furthermore, poor wellbeing suggests reduced employability and perhaps increased reliance on government benefits. Admittedly, these are difficult to calculate because many physical and mental health issues can be precursors to much more disabling disorders in later life. Recent estimates suggest that excessive body weight among children and young people is likely to be costly. For example, a report on obesity in Australia concluded that, after accounting for direct health costs, productivity losses and career costs, the value of a statistical life year, and other financial costs, the economic impact of obesity was estimated to be close to AUD 60 billion in 2008 alone [29].

The aforesaid health and mental health issues among children and young people can have considerable social implications. These include the oft-cited burden of care among family members [30,31,32,33,34], limited educational opportunities [35] and fewer employment prospects [9].

This paper explores the obesity ”epidemic” [6] among Australian young people. Using the national panel dataset of the Household, Income and Labour Dynamics in Australia (HILDA) Survey, this work examines potential differences in the impacts of economic, social, demographic and psychological factors by gender on obesity in young Australian adults. If there are gender differences in the conditioning of obesity, this will have implications for policies designed to combat the incidence of obesity among young adults. It is possible that such gender differences exist. There is a study which links the risk of anorexia nervosa to gender; see for example Lindberg and Hjern, who show that the risk is significantly higher for females [36]. In terms of the HILDA dataset used in this paper, the unconditional probability, over all years of the dataset, of being overweight/obese is 0.53 for young females, but 0.63 for young males. The next section gives a description of the data used in the paper, along with summary statistics. A third section deals with the empirical model and the test for gender differences in the impact of conditioning variables on obesity. This is followed by an analysis of the estimates and tests and the paper ends with some concluding remarks in terms of the implications of the results for policy.

## 2. Data Description and Summary Statistics

The data used for this study is the HILDA Survey. It is a household-based panel study that began in 2001 and is at its tenth wave, as at 2010. It includes a self-completion questionnaire (SCQ) that is provided to all individuals in the households aged 15 years and over. Since 2006, the SCQ has included additional questions on height and weight, permitting the calculation of body mass index (BMI). BMI is categorised as follows—underweight (BMI < 18.5); normal (18.5 ≤ BMI < 25.0); overweight (25.0 ≤ BMI < 30.0); and obese (BMI ≥ 30.0). In this paper, the dependent variable is dichotomous, scoring 1 if overweight/obese and 0 if normal. The underweight category is not included, as the focus is the conditioning of the probability of being overweight/obese and the proportion of young people underweight was very low (50 out of 6875). The overweight and obese are grouped together as there is a significant body of evidence across pediatric and adult populations demonstrating multiple health risks related with overweight and obesity [37].

This paper uses the longitudinal data from waves six to ten of HILDA—that is, years 2006 to 2010 inclusive—to evaluate gender and the conditioning factors of obesity for young males and females aged 15 to 24, which accords with the Australian government definition of young people [38,39].

Reading down the columns in Table 1 gives the BMI category in 2010 and reading across the rows gives the category in 2006. The elements on the principal diagonal give the probability of remaining in the same category; these are relatively large, indicative of stability over time. The off-diagonal elements give the probability of transition between categories. Over the five-year period, 13.8% of females and 14.7% of males who were within normal range moved to being overweight and obese. Almost 16% of young people undertook the reverse transition during the same period, but with more females than males.

Table 2 presents the description of and the descriptive statistics for the variables in the model. Individual-level variables are included that contain general information on age, gender, employment status, highest level of education attained, household characteristics, socio-economic status, marital status and geographical location. Additionally, expenditure on meals outside the home, alcohol consumption, and five personality traits (associated with the FFM) are included as measures of health status and personality of respondents.

Age is expected to have a non-linear association with BMI. To account for this, the variables *age* (in years) and *age*^2^ (*age* squared) are included in the model. The average age of respondents was about 20 years. The covariate *advantage*, the Socio-Economic Indexes for Areas (SEIFA) 2001 decile of index of relative socio-economic advantage/disadvantage [40], is included in this model to capture likely association between being in relatively low socio-economic status with higher overweight risk.

A significant relationship between level of education and obesity has been shown in many studies [41,42] where a low level of education attainment was found to be the strongest predictor of overweight/obese in both genders. The variable *educ*—the highest level of education attained by participants—is included in this model to capture this relationship. *Educ* comprised four ordered categories of educational attainment. *Area* is a categorical ordinal variable reported in HILDA and is included to capture the possibility of those living in a remote area having a higher BMI [43]. For the purposes of this analysis, employment status, *empstatus*, is re-coded from the HILDA data as a binary variable to indicate employed versus unemployed in the labour force.

Consumption spending on alcohol (*alcohol*) and on foods prepared outside the home (*meals*) is generally associated with increased obesity [44,45]. Potential differences in terms of varying household types are controlled by the inclusion of the variables *marstatus* and *hhtype*. Among adults, being married is associated with an increased risk of overweight and this relationship may also differ between males and females [45]. *hhtype* is a binary variable which isolates single parents with dependent children.

The main household income measure is real household annual disposable income. Household annual disposable income is the combined income of all household members after receipt of government pensions and benefits and deduction of income taxes in the financial year ended 30 June of the year of the wave. This is then adjusted for inflation—the rise in the general price level in the economy—using the Australian Bureau of Statistics Consumer Price Index—so that income is expressed in 2010 prices. Income is captured by the variable *lndinc*/*p*, which is household real annual disposable income scaled by the number of persons in the household who are included in the survey at the time the data was collected. The covariate *losat*, satisfaction with life, was collapsed from ten to three categories, especially to re-categorize those who rated themselves as dissatisfied with their life.

The five personality traits associated with the FFM are included as measures of health status and personality of respondents. The panel on obesity runs from year 2006 to 2010 inclusive. Personality data was collected for years 2005 and 2009. Personality scores are relatively stable and scores for year 2005 were applied to years 2006 and 2007, while scores for 2009 were applied to years 2008 and 2010 to complete the panel.

The FFM is well established in psychology literature [46], although used less frequently in econometric work [47]. The five personality traits: *opene*—openness to experience; *consc*—conscientiousness; *extrv*—extraversion; *agree*—agreeableness; and *emote*—emotional stability—are coded from 1 to 7 in HILDA from a factor analysis based on a set of 30 questions posed to participants. Low scores reflect the negative aspect of the traits and high scores reflect the positive aspect [46]. Some work has been done linking personality traits and obesity [48,49,50]. Rydén et al. used different personality variables to the FFM, namely the Karolinska Scales of Personality (KSP), and Sullivan et al. used the Temperament and Character Inventory (TCI). Fortunately, the TCI can be linked to the FFM [51].

The mean and standard deviation scores for the continuous variables in Table 2—*age*, *lndipinc*/*p*, *alcohol* and *meals*—have their usual meaning. The personality scores are ordinal, but take on 36 different ranks between the values 1 and 7 inclusive; as such, the reported means and standard deviations have the usual interpretation. The mean for the binary variables *gender*, *empstatus*, *hhtype* and *marstatus* give the proportion of the estimation sample scoring 1. The means and standard deviations for the remaining variables, which are all ordered categorical, should be interpreted with caution. The relative frequency distributions for these categorical variables are given in Table 3.

Also in Table 3, *advantage* is the SEIFA index, which is simply the decile of socio-economic advantage from the lowest to the highest ten percent. The fact that the relative frequencies all approximate to the value ten gives an indication of the representativeness of the HILDA sample.

## 3. The Empirical Model

In panel studies, random effects logit models are commonly used to estimate the subject-specific effects to accommodate unobserved individual heterogeneity. The distribution of a binary response variable, $y_{it}$, is described by the conditional probability of success.

$P(y_{it}\;=\;1|x_{it},\beta ,\alpha_{i})$, which is specified as an independent Bernoulli distribution conditional on the vector of variables **x***_it_*, the individual specific component α and a vector of parameters, **β**, to be estimated. In the random effects specification, the individual heterogeneity is a random variable, with density α*_i_* ~ *g*(α*_i_*).

For the logit, the conditional probability is [52]:

(1) $$P(y_{it}=1|x_{it},\alpha_{i})=\Lambda (\alpha_{i}+x_{it}^{\prime }\beta )$$

where Λ is the cumulative logit distribution, with a linear index $\sum_{j-1}^{J}\beta_{j}x_{jit}$.

The random effects specification may be consistently estimated using the likelihood criterion, with sample log likelihood [53]:

(2) $$L\left(x_{it},\alpha_{i},\beta |y_{it}\right)=\sum_{i=1}^{N}\ln\left(\int_{\infty }^{\infty }\left[\prod_{t=1}^{t=T}\Lambda {\left(\alpha_{i}+x_{it}^{\prime }\beta \right)}^{y_{it}}\left(1-\Lambda {\left(\alpha_{i}+x_{it}^{\prime }\beta \right)}^{1-y_{it}}\right)\right]g\left(\alpha_{i}\right)d\alpha_{i}\right)$$

where the density *g*( ) is designated standard normal.

The question is, how does one incorporate gender-specific differences in the conditioning of probability *P*, by the vector of variables x? In linear models, this issue is easily addressed by the use of interactive variables. However, the estimated coefficients of the standard binary model do not lend themselves to the same easy interpretation as the linear model [54]. Although it is possible to retrieve impacts and elasticities, there are some problems when interactions involve the interaction between discrete and continuous variables, such as gender, a discrete variable and say age or age squared, as continuous variables, where the marginal effect of the interaction would not be defined.

Alternatively, the linear index of conditioning variables and associated parameters of (1), **x’***_it_***β**, can be modified to accommodate gender-specific differences in the conditioning of probability by the vector **x**. That is:

(3) $$P\left(y_{it}=1|x_{it},\alpha_{i}\right)=\Lambda \left(\alpha_{i}+\left(1-I_{i}\right)x_{it}^{\prime }\beta_{F}+Ix_{it}^{\prime }\beta_{M}\right)$$

where *I* is an indicator with value 1 if the observation is male and 0 if the observation is female. Specification (1) is nested within (3) so that a likelihood ratio can be used to discriminate between the null of no gender difference in model structure and the alternate of (3).

The sign of the estimated coefficients can be used to show their effect on probability and standard tests can determine if variables are significant in determining probability. Alternatively, the odds ratio can be used to assess the impact of a change in the conditioning variable on probability. The odds ratio is defined as [55]:

(4) $$\frac{P\left(y_{it}=1|x_{it}\alpha_{i}\right)}{P\left(y_{it}=0|x_{it}\alpha_{i}\right)}=\frac{\exp\left(\alpha_{i}+x_{it}^{\prime }\beta \right)/1+\exp\left(\alpha_{i}+x_{it}^{\prime }\beta \right)}{1-\exp\left(\alpha_{i}+x_{it}^{\prime }\beta \right)/1+\exp\left(\alpha_{i}+x_{it}^{\prime }\beta \right)}=\exp\left(\alpha_{i}+x_{it}^{\prime }\beta \right)$$

Thus, a one-unit increase in variable **x***_j_* multiplies the initial odds ratio by exp(**β***_j_*). That is, if **β***_j_* = 1 then a one-unit increase in **x***_j_* multiplies the odds ratio by 2.7183, so that the relative probability increases by 171.83%.

The strategy adopted in this paper is to estimate model (3). Model (1) is then discriminated by applying a likelihood ratio test, with (1) as the restricted model. The next section deals with the outcomes of this strategy.

## 4. Results and Discussion

The previous section set up two rival empirical models of those factors conditioning youth obesity. Model (3) is an unrestricted model where the same variables condition the probability of being overweight/obese differently for females and males. Nested within this is model (1), where the probability conditioning is restricted to be the same for females and males. A simple way to discriminate between these two models is a likelihood ratio test with model (1) as the null hypothesis and model (3) as the alternate. The result of this test is given in Table 4, which shows that the null (model (1)) can be rejected at the 10% level. That is, there are significant differences by gender in the way that the candidate variables condition the probability of being overweight/obese.

The results for the random effects logit model (3) described in the previous section are presented in Table 5, with the estimated coefficient standard errors and odds ratio (OR) by gender. Before dealing with the individual variables and the differences between males and females, it would be useful to look at some summary statistics and tests contained in the header and footer of the table. The header gives the Wald statistic for the restriction that all slope coefficients equal zero. This restriction is rejected. The statistic σ^2^_α_ in the footer is the variance of the unobserved individual (random) effect. The variance of the idiosyncratic error is σ^2^_ε_. The ratio ρ, which is restricted to the unit interval, gives the proportion of total variance given by unobserved individual effect and is the correlation of overweight/obesity over time for individuals; it is indicative of the level of persistence in overweight/obesity for individuals [56,57]. Here the score, at 0.844, is close to 1, indicative that overweight/obesity is persistent for individuals over time. This confirms the validity of the estimation method, because 84.4% of variation in obesity is explained by the random effect and the likelihood ratio test with a *p*-value of 0.000 implies that the data fits the model well. Furthermore, Hausman Test was conducted to validate the Random Effect (RE) model against the Fixed Effect (FE) model.

Table 5 shows that there is a difference in conditioning of probability by gender. The signs of all the parameter estimates are as anticipated. Four of the independent variables are significant at the 1% level with a further variable significant at the 10% level for females. Two of these variables are not significant for males, *age* and *opene*. In contrast, for males there are only three variables significant at the 1% level, but with an additional four significant at 10%. Moreover, four of these variables are not significant for females, notably *area*, *meals*, *lndinc*/*p* and the personality characteristic extraversion, *extrv*.

The potential non-linear nature of the relationship between *age* and the probability of being overweight/obese is included in the model with the square of age, *age*^2^. Interestingly, even though the sample is restricted to young people, both age and square of age are statistically significant for females, with the probability of moving to the overweight/obese category increasing with age. This effect also decreases with age, because of the negative coefficient for *age*^2^.

The SEIFA index of disadvantage, variable *advantage*, is in integer-ordered score from 1 to 10 with 1 the least advantaged as detailed earlier. This variable is negatively significant for females, so that the probability of being overweight/obese decreases with increasing socio-economic advantage. Moving up the SEIFA index by one score reduces the odds of being overweight by 18% (18% ≈ 100 × (0.819–1). Remarkably, educational attainment, *educ*, is not significant for either gender.

The variable *area* measures remoteness in terms of population density, where 1 denotes being in a major city and 4 denotes remoteness. Male rural residents show a significant disadvantage in terms of obesity status, with a higher probability of being obese compared to their urban counterparts. In terms of percentage change in the estimated odds ratio, conditional odds increase by 54% for males with a single rank increase in remoteness.

Employment and alcohol consumption did not significantly affect probability of obesity for both genders. Due to its high calorie content, alcohol consumption is positively associated with obesity. According to Lukasiewicz et al., moderate alcohol drinkers tend not to gain weight relative to non-drinkers [58]. Arif and Rohrer found that those who drink moderately had lower odds of obesity as compared to non-drinkers, but the odds of overweight and obesity were significantly greater among binge drinkers and those consuming four or more drinks per day [59]. However, this appears not to be the case for young adults. The outcome for *meals* appears to be asymmetric, significant for males (at the 10% level) but not significant for females. However, the odds ratio for both females and males is 1: that is, the odds of being overweight/obese are unaffected by expenditure on meals outside the home.

The variable *marstatus* is binary, scoring 0 for single and 1 for being married or in a de facto relationship, as detailed previously. This variable is statistically significant and the probability of being overweight/obese is higher for those who are married or in a de facto relationship for both genders. A rationale for this may be in terms of marriage markets—once married, competition for a partner ceases and individuals become less concerned with appearance: that is, marriage causes overweight. The odds of being overweight/obese are approximately 0.37 (i.e., 1/2.699) and 0.43 times higher for females and males respectively who are married or de facto [60]. There is little difference in the odds ratio between males and females for marriage market effects. Being a single parent with dependent children is not a significant determinant of being overweight/obese, irrespective of gender. Disposable income is not significant for females, but it is for males, albeit at the 10% level, with probability increasing. This result for males is counterintuitive.

Finally, outcomes for personality characteristics, which are coded from 1 to 7 in HILDA, and low scores reflect that the negative aspects of personality traits are relatively asymmetric. Agreeableness and emotional stability are not significant for females and males. Conscientiousness is significant for both, at the 1% level for females and 10% for males. The negative sign is expected and replicates outcomes for the community sample in Sullivan et al. [50]. Extraversion is not significant for females but is for males at the 1% level. Sullivan et al. showed that being obese was positively associated with novelty seeking and reduced reward dependence, which is parallel with the positive sign for extraversion here [50]. Finally, openness to ideas is significant at the 10% level for females, indicating that those who are more open to new ideas are less likely to be overweight/obese.

Since it was rejected, detailed comments will not be made on the results for model (1) at this stage. However, the estimated coefficients, standard errors and odds ratios can be found in Table 6. Comparison of Table 6 with Table 5 indicates that the model is robust to variable exclusion.

A limitation of this study is the over-reliance on self-reported weight and height by individuals. It is recognized that people do not answer honestly about their weight or height and these inaccurate answers affect BMI calculation [61]. However, it is also known that while people may not answer honestly about their weight, there is a high correlation between reported and measured weight [24].

## 5. Conclusions

This paper has examined the factors influencing the probability of being overweight/obese in Australian young people. While there is evidence that demographic, economic, social and personality traits condition the incidence of obesity, there are significant differences between males and females in that conditioning. Age and having personality of openness were identified to be significant for females but not for males. While for males, area that they live in, meals eaten out, disposable income and being extravert were found to be statistically significant but not for females. As a generalization, the study identifies almost twice as many variables conditioning the incidence of obesity among females compared to males. These differences may have policy implications, even to the extent that a policy aimed at reducing the problem of obesity might not work if it fails to take gender differences into account.

## Figures and Tables

**Table 1:** Transition probabilities: young people (aged 15–24) (2006 to 2010).

	Obese 2010	
	0	1
	0.8580	0.1420
Obese	(0.8622)	(0.1378)
2006	[0.8534]	[0.1466]
	0.1574	0.8426
	(0.1574)	(0.8426)
	[0.1574]	[0.8426]

(Parentheses—females) [Brackets—males].

**Table 2:** Description and Descriptive Statistics for the Covariates Used in the Model.

Covariates Name	Description	Mean (Std. Dev.)		
		Male	Female	Total
*obese*	Body mass index group; normal = 0, overweight and obese = 1	0.363	0.354	0.358
		(0.481)	(0.478)	(0.480)
*gender*	Gender; male = 1, female = 0	1	0	0.481
		(0)	(0)	(0.50)
*age*	Age in years	19.885	20.018	19.954
		(2.551)	(2.526)	(2.539)
*advantage*	SEIFA 2001 deciles of index of relative socio-economic advantage/disadvantage; lowest = 1, highest = 10	5.765	5.654	5.707
		(2.867)	(2.860)	(2.863)
*educ*	Highest education level achieved; postgraduate/bachelor = 1, adv. Diploma/certificate = 2, year 12 = 3, year 11 and below = 4	3.103	2.946	3.021
		(0.873)	(0.960)	(0.922)
*area*	Remoteness area; major city = 0, inner regional Australia = 1, outer regional Australia = 2, remote Australia = 3, very remote Australia = 4	0.490	0.484	0.487
		(0.780)	(0.734)	(0.756)
*empstatus*	Labour force status; employed = 1, unemployed and not in the labor force = 0	0.743	0.713	0.728
		(0.437)	(0.452)	(0.445)
*alcohol*	Drink alcohol; no = 0, yes = 1	0.840	0.819	0.829
		(0.367)	(0.385)	(0.377)
*meals*	Household annual expenditure on meals eaten out (AUD)	2682.87	2775.56	2731.01
		(2520.61)	(3487.80)	(3061.37)
*marstatus*	Marital status; married or in a de facto relationship = 1, single = 0	0.178	0.262	0.221
		(0.382)	(0.440)	(0.415)
*hhtype*	Household type; lone parent with children <15 and lone parent with dependents = 1, others = 0	0.044	0.052	0.048
		(0.205)	(0.222)	(0.214)
*inc/p*	Household disposable income divided by the number of persons in households	26,099	26,063	26,080
		(13,653)	(14,988)	(14,358)
*losat*	Satisfaction with life; dissatisfied = 1, neutral = 2, satisfied = 3	2.912	2.900	2.905
		(0.301)	(0.323)	(0.312)
*agree*	Personality scale—Agreeableness (1 to 7)	4.929	5.393	5.170
		(0.963)	(0.852)	(0.936)
*consc*	Personality scale—Conscientiousness (1 to 7)	4.565	4.764	4.668
		(0.970)	(1.008)	(0.995)
*emote*	Personality scale—Emotional stability (1 to 7)	5.023	4.773	4.893
		(1.040)	(1.052)	(1.054)
*extrv*	Personality scale—Extroversion (1 to 7)	4.583	4.708	4.648
		(0.951)	(1.049)	(1.005)
*opene*	Personality scale—Openness to experience (1 to 7)	4.247	4.338	4.294
		(1.049)	(1.042)	(1.046)

**Table 3:** The Frequency Distribution of Categorical Variables (%).

Categories/Variables	Advantage		Education		Area		Life Satisfaction	
	Male	Female	Male	Female	Male	Female	Male	Female
0					65.61	64.41		
1	7.71	9.21	6.01	10.31	22.42	24.13	0.51	0.68
2	9.18	9.89	15.42	17.94	10.08	10.25	7.77	8.71
3	11.32	9.15	40.85	38.58	1.15	1.04	91.71	90.61
4	8.64	9.12	37.72	33.17	0.74	0.18		
5	9.72	9.51						
6	9.53	9.86						
7	9.60	10.99						
8	10.56	11.37						
9	12.92	11.16						
10	10.81	9.74						

**Table 4:** Likelihood-Ratio Test (LR) Unrestricted and Restricted Models by Gender.

H_0_: $P\left(y_{it}=1|x_{it},\alpha_{i}\right)=\Lambda \left(\alpha_{i}+x_{it}^{\prime }\beta \right)$ (1)	
H_1_: $P\left(y_{it}=1|x_{it},\alpha_{i}\right)=\Lambda \left(\alpha_{i}+\left(1-I_{i}\right)x_{it}^{\prime }\beta_{F}+I_{i}x_{it}^{\prime }\beta_{M}\right)$ (3)	
LR χ^2^ (18) = 27.81	Pr > χ^2^ (18) = 0.065

**Table 5:** Unrestricted Model, Coefficients, Standard Errors and Odds Ratios (OR).

$P\left(y_{it}=1|x_{it},\alpha_{i}\right)=\Lambda \left(\alpha_{i}+\left(1-I_{i}\right)x_{it}^{\prime }\beta_{F}+I_{i}x_{it}^{\prime }\beta_{M}\right)$ (3)						
N = 6503						
Log Likelihood = −3115.66, Wald χ^2^ (36) = 452.86, Pr > χ^2^ = 0.000						
	**Female**			**Male**		
**Variable**	**Coef.**	**Std. Err.**	**OR**	**Coef.**	**Std. Err.**	**OR**
*constant*	−24.324 ***	6.772		−17.025 ***	6.964	
*age*	2.353 ***	0.622	10.522	0.956	0.638	2.600
*age*^2^	−0.050 ***	0.015	0.951	−0.015	0.016	0.985
*advantage*	−0.200 ***	0.044	0.819	−0.078 *	0.046	0.925
*educ*	−0.008	0.144	0.992	0.062	0.152	1.064
*area*	0.247	0.172	1.280	0.433 ***	0.167	1.542
*empstatus*	−0.336	0.213	0.714	0.054	0.228	1.056
*alcohol*	−0.061	0.239	0.941	0.062	0.285	1.064
*meals*	−5.1 × 10^−5^	3.2 × 10^−5^	1.000	−3.07 × 10^−6^ *	3.55 × 10^−5^	1.000
*marstatus*	0.993 ***	0.261	2.699	0.844 ***	0.303	2.327
*hhtype*	0.209	0.438	1.232	0.430	0.476	1.537
*lndinc/p*	0.064	0.181	1.066	0.306 *	0.186	1.358
*losat*	−0.327	0.272	0.721	−0.177	0.306	0.837
*agree*	0.097	0.147	1.102	−0.108	0.137	0.898
*consc*	−0.432 ***	0.121	0.649	−0.222 *	0.135	0.801
*emote*	0.065	0.123	1.068	−0.137	0.125	0.872
*extrv*	−0.103	0.119	0.902	0.372 ***	0.132	1.451
*opene*	−0.254 *	0.131	0.75	−0.006	0.136	0.994
lnσ^2^_α_	2.878	0.091				
σ_α_	4.217	0.192				
$\rho =\frac{\sigma_{\alpha }^{2}}{\sigma_{\alpha }^{2}+\sigma_{\varepsilon }^{2}}$	0.844	0.012				

* 10% significance, *** 1% significance.

**Table 6:** Restricted Model, Coefficients and Standard Errors.

$P\left(y_{it}=1|x_{it},\alpha_{i}\right)=\Lambda \left(\alpha_{i}+x_{it}^{\prime }\beta \right)$ (1)		
N = 6503		
Log Likelihood = −3129.57 Wald χ^2^ (17) = 231.93 Pr > χ^2^ = 0.000		
**Variable**	**Coef.**	**Std. Err.**
*constant*	−21.507 ***	4.848
*age*	1.737 ***	0.444
*age*^2^	−0.034 ***	0.011
*advantage*	−0.146 ***	0.032
*educ*	0.046	0.104
*area*	0.344 ***	0.121
*empstatus*	−0.143	0.155
*alcohol*	−0.012 *	0.183
*meals*	−2.9 × 10^−5^	2.25 × 10^−5^
*marstatus*	0.926 ***	0.197
*hhtype*	0.314	0.321
*lndinc/p*	0.185	0.130
*losat*	−0.254	0.203
*agree*	−0.004	0.098
*consc*	−0.303 ***	0.089
*emote*	−0.001	0.087
*extrv*	0.088	0.088
*opene*	−0.121	0.094
lnσ^2^_α_	2.901	0.091
σ_α_	4.265	0.194
$\rho =\frac{\sigma_{\alpha }^{2}}{\sigma_{\alpha }^{2}+\sigma_{\varepsilon }^{2}}$	0.847	0.012

* 10% significance, *** 1% significance
